# Drug-coated balloon angioplasty versus conventional angioplasty for symptomatic non-acute intracranial artery occlusion

**DOI:** 10.3389/fneur.2025.1570728

**Published:** 2025-08-15

**Authors:** Kaiyue Dong, Yingkun Chen, Yun Song, Lili Sun, Meimei Zheng, Hao Yin, Jun Zhang, Shuoshuo Li, Tianxi Yin, Wei Wang, Yao Meng, Weili Li, Xiao He, Chenlu Zhu, Wei Zhao, Ju Han

**Affiliations:** Department of Neurology, The First Affiliated Hospital of Shandong First Medical University and Shandong Provincial Qianfoshan Hospital, Jinan, China

**Keywords:** intracranial atherosclerotic occlusion, drug-coated balloon, angioplasty, restenosis, outcome

## Abstract

**Background and objective:**

Non-acute intracranial artery occlusion (ICAO) is an important cause of recurrent ischemic stroke despite aggressive medical treatment. Conventional angioplasty has high incidences of perioperative complications and restenosis in treating these patients. Drug-coated balloon (DCB) treatment has shown superiority in preventing restenosis. This study aimed to compare the DCB angioplasty with conventional angioplasty in symptomatic ICAO patients.

**Methods:**

From January 2016 to March 2024, consecutive patients with symptomatic non-acute ICAO treated with endovascular therapy were enrolled and dichotomized by whether DCB was used. Propensity score matching analysis was used to compare the perioperative complications, clinical and imaging follow-up results of the two groups.

**Results:**

158 eligible patients were included in this study. There were 104 cases in the DCB group and 54 cases in the non-DCB group. A total of 92 matched patients were selected for propensity score matching analysis. At 6 months follow-up, the median stenosis degree (19.3% [0% ~ 34.3%] vs. 43.6% [22.8% ~ 80.2%], *p* = 0.009) and total restenosis incidence (10.9% [5/46] vs. 39.1% [18/46], *p* = 0.004) were significantly lower in the DCB group. There were no statistically significant differences in the incidences of perioperative complications (8.7% vs. 17.4%, *p* = 0.388), symptomatic restenosis (2.2% vs. 8.7%, *p* = 0.375) and recurrent ischemic events (4.3% vs. 10.9%, *p* = 0.453) between the two groups.

**Conclusion:**

In patients with symptomatic non-acute ICAO, compared with conventional angioplasty, DCB angioplasty can effectively reduce the restenosis degree and restenosis risk.

## Introduction

Non-acute intracranial artery occlusion (ICAO) is one of the important causes of recurrent ischemic stroke despite aggressive medical treatment, and seriously threatens human life and health (1–3). The optimal treatment for these patients is not well-established. In recent years, with the development of interventional technology, angioplasty has gradually become an important method for the treatment of non-acute symptomatic ICAO (4–8). However, the incidence of perioperative complications and restenosis risk after conventional angioplasty is high, which increases the risk of stroke recurrence in occlusive patients.

Drug-coated balloon (DCB) is coated with antiproliferative drugs to play the role of inhibiting intimal hyperplasia and preventing restenosis. When DCB dilates, the antiproliferative drugs can be penetrated from balloon surface into the vessel wall. In recent years, DCB has been gradually applied to the treatment of symptomatic non-acute ICAO to reduce restenosis (9). Our preliminary study has shown the feasibility of DCB in the treatment of ICAO, with a lower incidence of restenosis (9). But there is no study to compare DCB treatment with conventional angioplasty for non-acute ICAO. This study aimed to assess the safety and efficacy of DCB treatment (with or without stenting) compared with conventional angioplasty in symptomatic non-acute ICAO.

## Methods

### Study population

We retrospectively enrolled patients with symptomatic non-acute ICAO who received endovascular interventions from January 2016 to March 2024. Patients initially diagnosed ICAO by CT angiography (CTA) or magnetic resonance angiography (MRA) and confirmed by digital subtraction angiography (DSA). The inclusion criteria were as follows: 1. occlusion time> 24 h (occlusion time is defined as the time from initial radiological diagnosis to endovascular treatment); 2. Intracranial atherosclerosis was the primary etiology; 3. recurrent transient ischemic attack (TIA) or stroke associated with intracranial artery occlusion despite aggressive medical management, including dual antiplatelet therapy (DAPT), statins, and lifestyle interventions; 4. total occlusion in the intracranial internal carotid artery (Intra ICA), middle cerebral artery (MCA), intracranial vertebral artery (Intra VA), or basilar artery (BA); 5. vascular imaging follow-up using DSA was performed after treatment. Exclusion criteria are as follows: 1. non-atherosclerotic diseases such as suspected cerebral vasculitis, arterial dissection, and cardiac embolism; 2. The target artery has tandem multiple stenosis lesions. Based on whether DCB was used or not, these patients were further divided into DCB and non-DCB groups. Informed consent was obtained from all patients or their authorized family members before surgery, and this study was approved by the review committee of our hospital.

### Procedures

The details of interventional procedures have been described in previous studies (10, 11). DAPT (aspirin 100 mg/day and clopidogrel 75 mg/day) was given at least for 5 days before procedure. The procedures were performed under general anesthesia.

For the DCB group, adequate pre-inflation with a conventional balloon must be performed prior to DCB (SeQuent Please; B. Braun, Berlin, Germany) dilation. The DCB needed to cover the entire lesion and inflated slowly for 60 s at nominal pressure to deliver paclitaxel to the vessel wall. After the DCB was removed, angiography was performed again. If residual stenosis was > 50% or if there was vascular dissection after DCB dilation, salvage stenting is performed.

For the non-DCB group, the target vessel was dilated by the conventional balloon. Angiography was performed to assess the lumen after adequate dilation of the conventional balloon, and salvage stenting was performed if the residual stenosis was > 50% or if there was vascular dissection after conventional balloon dilation.

The choice of stent for these patients was left to the discretion of the operator. All stents were conventional stents. DAPT was maintained for 3 months in patients without stenting (DCB dilation or conventional balloon dilation), and 6 months in patients with stenting implantation. Then the patients were treated with Aspirin or clopidogrel monotherapy thereafter.

### Data collection and follow-up outcomes

Demographic, angiographic, clinical, and perioperative data were collected. All patients were followed up at 1 month, 3 months, 6 months, 1 year, and annually thereafter. They were scheduled to perform DSA at 6 months (±1 month) after the interventional procedure. The primary follow-up outcomes were angiographic restenosis, recurrent ischemic events, and symptomatic restenosis at 6-month follow-up. Restenosis was defined as > 50% stenosis within or immediately adjacent (within 5 mm) of the treated segment and >20% absolute lumen loss (12). Recurrent ischemic events were defined as focal neurological symptoms related to the corresponding vascular territory. Symptomatic restenosis was defined as restenosis associated with ischemic symptoms of the offending vessel territory. 2 investigators reviewed imaging and clinical results and differences were resolved by consensus.

### Statistical analysis

SPSS version 26.0 for Windows (IBM, Armonk, New York) was used for statistical analysis. Continuous variables were expressed as the median with interquartile ranges, or as the mean ± standard deviation (SD) and compared using the Student *t*-test or Mann–Whitney *U* test. Categorical variables were expressed as numbers and percentages and compared using a chi-square test or Fisher’s exact test. Propensity Score Matching (PSM) was used to compare the results of the two groups. For PSM, age and lesion length were used as covariates, and the caliper width of the propensity score was 0.02, and a 1:1 match was performed based on the nearest neighbor matching algorithm. After 1:1 matching, McNemar test was used for the categorical variables, and Wilcoxon signed-rank test or Paired Sample *t*-Test was used for the numerical variables to test the differences in baseline characteristics and outcomes.

## Results

### Study population

**Figure 1:**
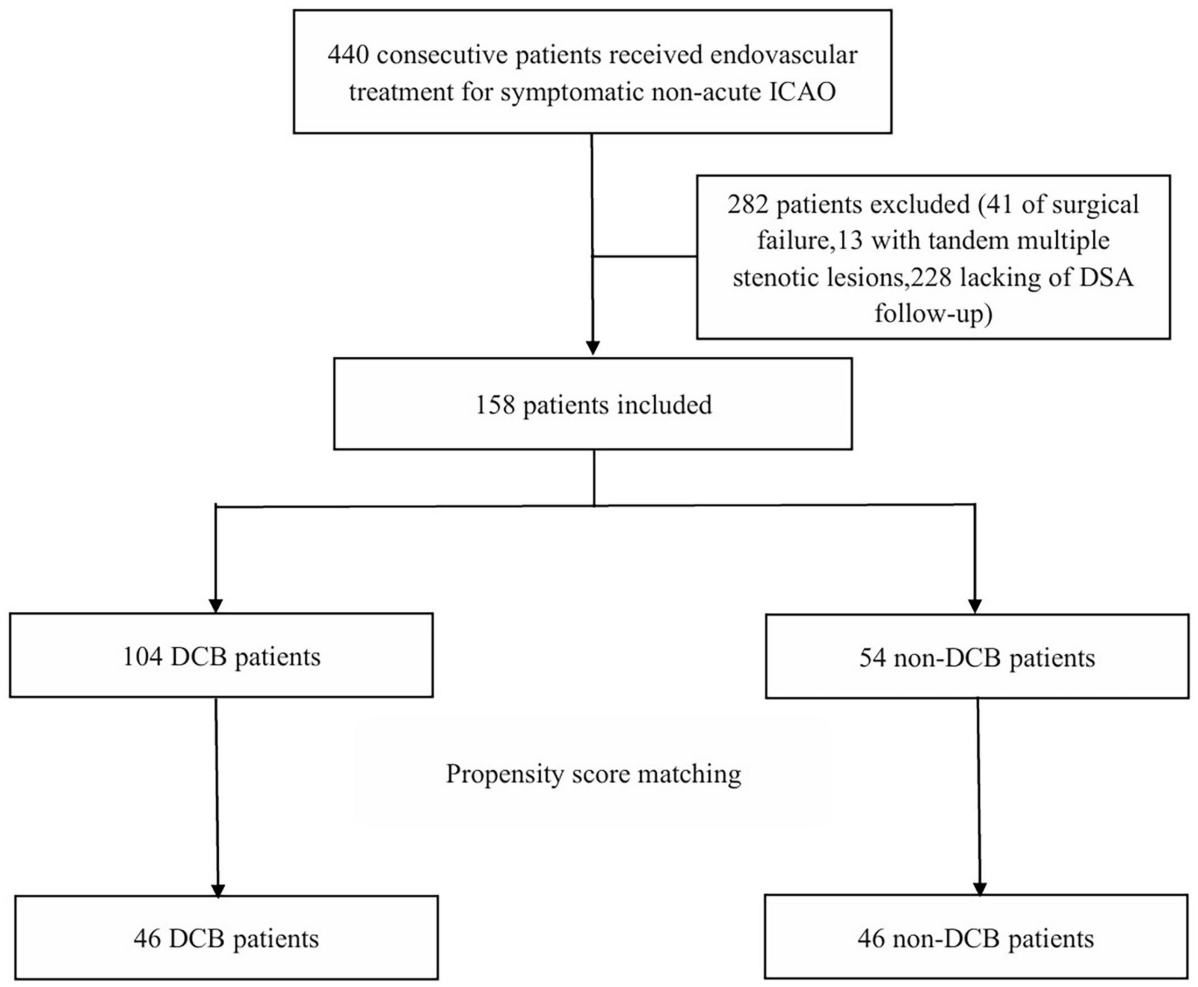
Patient flowchart. Flowchart of the study. DCB, drug-coated balloon; ICAO, intracranial artery occlusion; DSA, digital subtraction angiography.

A total of 158 consecutive patients received successful endovascular treatment for symptomatic non-acute ICAO with DSA follow-up were included between January 2016 and March 2024, including 104 in the DCB group and 54 in the non-DCB group. After PSM, 92 patients were finally matched, with 46 patients in each group. The patient flow chart was shown in Figure 1.

### Baseline, periprocedural, and outcome characteristics of the patients before PSM

The baseline, periprocedural, and outcome characteristics of the patients before PSM were shown in Table 1. There were no significant differences between the two groups in terms of age, gender, smoking history, hypertension, diabetes mellitus, hyperlipidemia, coronary heart disease, and ischemic stroke. There were no significant differences between the two groups in baseline NIHSS score (*p* = 0.991) and mRS score (*p* = 0.731). There were no significant differences between the two groups in occlusion to treatment time (d) (15.5 [8.0 ~ 29.8] vs. 15.5 [7.8 ~ 38.5], *p* = 0.841) and symptom onset to treatment time (d) (28.5 [18.3 ~ 58.8] vs. 26.0 [15.8 ~ 55.3], *p* = 0.680). There were 104 patients received DCB therapy (with or without stenting) in the DCB group, and 54 patients underwent balloon dilatation (with or without stenting) in the non-DCB group. Before surgery, there were no statistically significant differences in the HBALC (%) (6.10 [5.65 ~ 6.95] vs. 5.75 [5.60 ~ 6.45], *p* = 0.088) and LDL (mmol/L) (1.82 [1.39 ~ 2.23] vs. [2.02 ± 0.65], *p* = 0.203) between the two groups. All patients had good glycemic control and LDL control close to 1.8 mmoL/L.

**Table 1:** Baseline, periprocedural, and outcome characteristics of the patients.

Variables	All (*n* = 158)	DCB group (*n* = 104)	Non-DCB group (*n* = 54)	*p* value
Demographics				
Mean age ± SD, yrs	56.6 ± 10.0	57.4 ± 9.8	55.0 ± 10.4	0.164
Male, *n* (%)	107 (67.7)	70 (67.3)	37 (68.5)	0.877
Medical history				
Hypertension, *n* (%)	109 (69.0)	70 (67.3)	39 (72.2)	0.526
Diabetes mellitus, *n* (%)	52 (32.9)	36 (34.6)	16 (29.6)	0.527
Hyperlipidemia, *n* (%)	16 (10.1)	11 (10.6)	5 (9.3)	0.795
Coronary artery disease, *n* (%)	27 (17.1)	22 (21.2)	5 (9.3)	0.060
Ischemic stroke history, *n* (%)	34 (21.5)	22 (21.2)	12 (22.2)	0.877
Smoking, *n* (%)	70 (44.3)	47 (45.2)	23 (42.6)	0.755
Symptom onset to treatment (days), median (IQR)	28.0 (18.0–56.0)	28.5 (18.3–58.8)	26.0 (15.8–55.3)	0.680
Image-documented occlusion to treatment (days), median (IQR)	15.5 (8.0–31.3)	15.5 (8.0–29.8)	15.5 (7.8–38.5)	0.841
Pre-treatment NIHSS score, median (IQR)	3.0 (0.8–5.0)	3.0 (1.0–5.0)	3.0 (0–5.0)	0.991
Pre-treatment mRS score, median (IQR)	1.0 (1.0–3.0)	1.0 (1.0–3.0)	2.0 (1.0–3.0)	0.731
Occlusion location, *n* (%)				0.430
Anterior circulation	120 (75.9)	81 (77.9)	39 (72.2)	
Posterior circulation	38 (24.1)	23 (22.1)	15 (27.8)	
Periprocedural characteristics				
Occlusion length (mm), median (IQR)	10.7 (6.6–15.1)	9.0 (5.7–13.7)	12.5 (8.9–19.6)	0.002
Stenosis degree after intervention (%), median (IQR)	24.9 (0–35.0)	25.0 (0–35.5)	24.7 (0–33.6)	0.486
Periprocedural complications, *n* (%)	18 (11.4)	10 (9.6)	8 (14.8)	0.329
Symptomatic perioperative complications, *n* (%)	13 (8.2)	6 (5.8)	7 (13.0)	0.209
Follow-up outcomes				
Stenosis degree at follow-up (%), median (IQR)	27.9 (2.8–58.9)	24.5 (0–46.9)	39.7 (17.7–73.8)	0.015
Restenosis, *n* (%)	43 (27.2)	21 (20.2)	22 (40.7)	0.006
Symptomatic restenosis, *n* (%)	12 (7.6)	7 (6.7)	5 (9.3)	0.801
Recurrent ischemic events, *n* (%)	14 (8.9)	9 (8.7)	5 (9.3)	1.000
mRS score, median (IQR)	1.0 (0–2.0)	1.0 (0–2.0)	1.0 (0–2.0)	0.497

Values are mean ± SD or median (interquartile range), or *n* (%).

NIHSS, National Institutes of Health Stroke Scale; mRS, modified Rankin Scale; SD, standard deviation; IQR, interquartile range; DCB, drug-coated balloon.

Before PSM, lesion length (mm) was significantly different between the DCB group and the non-DCB group (9.0 [5.7 ~ 13.7] vs. 12.5 [8.9 ~ 19.6], *p* = 0.002). There were no significant differences in the incidence of anterior circulatory occlusion (77.9% vs. 72.2%, *p* = 0.430) and the degree of residual stenosis after intervention (25.0% [0% ~ 35.5%] vs. 24.7% [0% ~ 33.6%], *p* = 0.486). In terms of clinical and radiographic follow-up results, the stenosis degree (24.5% [0% ~ 46.9%] vs. 39.7% [17.7% ~ 73.8%], *p* = 0.015), the incidence of restenosis (20.2% [21/104] vs. 40.7% [22/54], *p* = 0.006) in the DCB group were significantly lower than those in the non-DCB group. There were no significant differences in perioperative complications (9.6% vs. 14.8%, *p* = 0.329), symptomatic perioperative complications (5.8% vs. 13.0%, *p* = 0.209), recurrent ischemic events (8.7% vs. 9.3%, *p* = 1.000), and symptomatic restenosis (6.7% vs. 9.3%, *p* = 0.801) between the two groups. The mRS score at 6 months postoperative follow-up was significantly lower than preoperative in the DCB group (1.0 [0 ~ 2.0] vs. 1.0 [1.0 ~ 3.0], *p* < 0.001) and the mRS score at 6 months postoperative follow-up was also significantly lower than preoperative in the non-DCB group (1.0 [0 ~ 2.0] vs. 2.0 [1.0 ~ 3.0], *p* < 0.001).

### Results of the patients after PSM

After PSM, the baseline balance between the two groups was good (Table 2). Among the 46 patients in the DCB group, 30 were treated with DCB dilation and 16 were treated with DCB dilation and salvage stenting. Among them, 13 patients underwent salvage stenting due to residual stenosis >50%, and 3 cases underwent salvage stenting due to vascular dissection after DCB dilation. Among the 46 patients in the non-DCB group, 25 were treated with conventional balloon dilation and 21 were treated with conventional balloon dilation and salvage stenting. Among them, 20 patients underwent salvage stenting due to residual stenosis >50%, and the other 1 case underwent salvage stenting due to vascular dissection after conventional balloon angioplasty. Angiography follow-up was completed at 6.0 months (IQR, 3.6–7.0 months). Median stenosis (19.3% [0% ~ 34.3%] vs. 43.6% [22.8% ~ 80.2%], *p* = 0.009), incidence of restenosis (10.9% [5/46] vs. 39.1% [18/46], *p* = 0.004) were significantly lower in the DCB group than those in the non-DCB group. However, although the absolute incidences of recurrent ischemic events (4.3% [2/46] vs. 10.9% [5/46], *p* = 0.453), symptomatic restenosis (2.2% [1/46] vs. 8.7% [4/46], *p* = 0.375) were lower in the DCB group than those in the non-DCB group, there were no statistically significant differences. The mRS score at 6 months postoperative follow-up was significantly lower than preoperative in the DCB group (1.0 [0 ~ 2.0] vs. 1.0 [1.0 ~ 2.3], *p* = 0.001) and the mRS score at 6 months postoperative follow-up was also significantly lower than preoperative in the non-DCB group (1.0 [0 ~ 2.0] vs. 2.0 [1.0 ~ 3.0], *p* < 0.001).

**Table 2:** Baseline, periprocedural, and outcome characteristics of the matched patients.

Variables	All (*n* = 92)	DCB group (*n* = 46)	non-DCB group (*n* = 46)	*p* value
Demographics				
Mean age ± SD, yrs	54.6 ± 10.1	54.9 ± 9.6	54.4 ± 10.7	0.735
Male, *n* (%)	63 (68.5)	32 (69.6)	31 (67.4)	1.000
Medical history				
Hypertension, *n* (%)	65 (70.7)	30 (65.2)	35 (76.1)	0.383
Diabetes mellitus, *n* (%)	26 (28.3)	12 (26.1)	14 (30.4)	0.791
Hyperlipidemia, *n* (%)	11 (12.0)	6 (13.0)	5 (10.9)	1.000
Coronary artery disease, *n* (%)	13 (14.1)	9 (19.6)	4 (8.7)	0.267
Ischemic stroke history, *n* (%)	22 (23.9)	10 (21.7)	12 (26.1)	0.815
Smoking, *n* (%)	44 (47.8)	24 (52.2)	20 (43.5)	0.503
Symptom onset to treatment (days), median (IQR)	28.0 (19.3–53.5)	28.5 (19.8–48.3)	27.0 (18.8–61.3)	0.922
Image-documented occlusion to treatment (days), median (IQR)	15.5 (9.0–33.8)	15.0 (8.8–29.8)	18.0 (8.8–44.8)	0.574
Pre-treatment NIHSS score, median (IQR)	3.0 (1.0–5.0)	2.0 (1.0–4.3)	4.0 (0–5.0)	0.553
Pre-treatment mRS score, median (IQR)	2.0 (1.0–3.0)	1.0 (1.0–2.3)	2.0 (1.0–3.0)	0.241
Occlusion location, *n* (%)				1.000
Anterior circulation	69 (75.0)	35 (76.1)	34 (73.9)	
Posterior circulation	23 (25.0)	11 (23.9)	12 (26.1)	
Periprocedural characteristics				
Occlusion length (mm), median (IQR)	11.7 (8.1–15.4)	11.9 (8.1–15.6)	11.4 (8.5–15.1)	0.942
Stenosis degree after intervention (%), median (IQR)	24.3. (0–33.1)	23.3 (0–32.4)	25.0 (3.0–34.7)	0.496
Periprocedural complications, *n* (%)	12 (13.0)	4 (8.7)	8 (17.4)	0.388
Symptomatic perioperative complications, *n* (%)	10 (10.9)	3 (6.5)	7 (15.2)	0.344
Follow-up outcomes				
Stenosis degree at follow-up (%), median (IQR)	27.3 (0.7–54.0)	19.3 (0–34.3)	43.6 (22.8–80.2)	0.009
Restenosis, *n* (%)	23 (25.0)	5 (10.9)	18 (39.1)	0.004
Symptomatic restenosis, *n* (%)	5 (5.4)	1 (2.2)	4 (8.7)	0.375
Recurrent ischemic events, *n* (%)	7 (7.6)	2 (4.3)	5 (10.9)	0.453
mRS score, median (IQR)	1.0 (0–2.0)	1.0 (0–2.0)	1.0 (0–2.0)	0.972

Values are mean ± SD or median (interquartile range), or *n* (%).

NIHSS, National Institutes of Health Stroke Scale; mRS, modified Rankin Scale; SD, standard deviation; IQR, interquartile range; DCB, drug-coated balloon.

## Discussion

A large number of symptomatic patients with non-acute ICAO remain hemodynamically unstable and present with recurrent ischemic events despite aggressive medical therapy (13). The CMOSS trial showed that the addition of bypass surgery to medical therapy did not significantly alter the risk of stroke or death within 30 days or the composite outcome of ipsilateral ischemic stroke within 30 days to 2 years in patients with symptomatic ICA or MCA occlusion and hemodynamic insufficiency (14). Medical therapy or bypass surgery plus medical therapy does not improve the outcome of medically refractory non-acute ICAO. There has been no consensus on the optimal treatment for these patients until now.

Previous studies showed that angioplasty was an important method for the treatment of symptomatic non-acute symptomatic ICAO (15–17). In this study, the mRS scores at 6 months follow-up were significantly lower than preoperative in the two groups, and the difference was statistically significant (*p* < 0.05). These patients experienced improved clinical outcomes after endovascular recanalization. In current interventional practice, restenosis after endovascular intervention remains a challenge. The incidence of restenosis is a major factor affecting the long-term outcome of endovascular intervention therapy (18). In recent years, with the development of interventional technology, DCB has been gradually applied to the treatment of symptomatic non-acute symptomatic ICAO to reduce the incidence of restenosis (9, 19, 20).

In this study, we compared the safety and efficacy of DCB angioplasty (with or without stenting) versus conventional angioplasty (with or without stenting) in patients with symptomatic non-acute ICAO. At 6 months follow-up, the median stenosis degree and total restenosis risk of DCB group were significantly lower than those in the non-DCB group. Although there were no statistically significant differences, the absolute incidence of recurrent ischemic events and symptomatic restenosis were lower in the DCB group than those in the non-DCB group.

The results of this study indicated that, this new strategy, DCB-directed angioplasty, may be an alternative treatment for symptomatic non-acute ICAO. However, this hypothesis still needs to be confirmed by large-scale, prospective, randomized controlled trials.

### Limitations

Our study has some limitations. First, this study was retrospective and might have selection bias despite propensity score matching analysis was applied to balance potential covariates. Second, the follow-up time point was 6 months, so the results should be extrapolated to long-term follow-up with caution.

## Conclusion

DCB angioplasty appears to be effective in reducing the degree of restenosis and the incidence of restenosis in patients with symptomatic non-acute ICAO. DCB angioplasty is a potential treatment for symptomatic non-acute ICAO. Further prospective randomized studies are needed to confirm these findings.

## Data Availability

The original contributions presented in the study are included in the article/supplementary material, further inquiries can be directed to the corresponding authors.

## References

[1] Wang Y Zhao X Liu L Soo YO Pu Y Pan Y. Prevalence and outcomes of symptomatic intracranial large artery stenoses and occlusions in China: the Chinese intracranial atherosclerosis (CICAS) study. Stroke. (2014) 45:663–9. doi: 10.1161/STROKEAHA.113.003508, PMID: 24481975

[2] Gorelick PB Wong KS Bae HJ Pandey DK. Large artery intracranial occlusive disease: a large worldwide burden but a relatively neglected frontier. Stroke. (2008) 39:2396–9. doi: 10.1161/STROKEAHA.107.505776, PMID: 18535283

[3] Yamauchi H Fukuyama H Nagahama Y Nabatame H Nakamura K Yamamoto Y. Evidence of misery perfusion and risk for recurrent stroke in major cerebral arterial occlusive diseases from PET. J Neurol Neurosurg Psychiatry. (1996) 61:18–25. doi: 10.1136/jnnp.61.1.18, PMID: 8676151 PMC486449

[4] Zhao W Zhang J Meng Y Zhang Y Zhang J Song Y. Symptomatic atherosclerotic non-acute intracranial vertebral artery total occlusion: clinical features, imaging characteristics, endovascular recanalization, and follow-up outcomes. Front Neurol. (2020) 11:598795. doi: 10.3389/fneur.2020.598795, PMID: 33312156 PMC7703109

[5] Zheng M Song Y Zhang J Zhao W Sun L Yin H. Endovascular recanalization of non-acute symptomatic middle cerebral artery Total occlusion and its short-term outcomes. Front Neurol. (2019) 10:484. doi: 10.3389/fneur.2019.00484, PMID: 31156533 PMC6529837

[6] Zhou C Cao YZ Jia ZY Zhao LB Lu SS Shi HB. Endovascular recanalization of symptomatic chronic ICA occlusion: procedural outcomes and radiologic predictors. AJNR Am J Neuroradiol. (2023) 44:303–10. doi: 10.3174/ajnr.A7804, PMID: 36822826 PMC10187816

[7] Yao YD Liu AF Qiu HC Zhou J Li C Wang Q. Outcomes of late endovascular recanalization for symptomatic non-acute atherosclerotic intracranial large artery occlusion. Clin Neurol Neurosurg. (2019) 187:105567. doi: 10.1016/j.clineuro.2019.105567, PMID: 31704389

[8] Zhao W Zhang J Song Y Sun L Zheng M Yin H. Endovascular recanalization for symptomatic subacute to chronic atherosclerotic basilar artery occlusion. Front Neurol. (2019) 10:1290. doi: 10.3389/fneur.2019.01290, PMID: 31920916 PMC6923246

[9] Zhao W Chu X Song Y Zhang J Sun L Zheng M. Drug-coated balloon treatment for delayed recanalization of symptomatic intracranial artery occlusion. Transl Stroke Res. (2023) 14:193–9. doi: 10.1007/s12975-022-01024-5, PMID: 35460456 PMC9995415

[10] Han J Zhang J Zhang X Zhang J Song Y Zhao W. Drug-coated balloons for the treatment of symptomatic intracranial atherosclerosis: initial experience and follow-up outcome. J Neurointerv Surg. (2019) 11:569–73. doi: 10.1136/neurintsurg-2018-014237, PMID: 30337378

[11] Zhang J Zhang X Zhang J Song Y Zheng M Sun L. Drug-coated balloon dilation compared with conventional stenting angioplasty for intracranial atherosclerotic disease. Neurosurgery. (2020) 87:992–8. doi: 10.1093/neuros/nyaa191, PMID: 32445576

[12] Derdeyn CP Fiorella D Lynn MJ Turan TN Cotsonis GA Lane BF. Nonprocedural symptomatic infarction and in-stent restenosis after intracranial angioplasty and stenting in the SAMMPRIS trial (stenting and aggressive medical Management for the Prevention of recurrent stroke in intracranial stenosis). Stroke. (2017) 48:1501–6. doi: 10.1161/STROKEAHA.116.014537, PMID: 28455321 PMC8204379

[13] Yamauchi H Higashi T Kagawa S Kishibe Y Takahashi M. Chronic hemodynamic compromise and cerebral ischemic events in asymptomatic or remote symptomatic large-artery intracranial occlusive disease. AJNR Am J Neuroradiol. (2013) 34:1704–10. doi: 10.3174/ajnr.A3491, PMID: 23471022 PMC7965647

[14] Ma Y Wang T Wang H Amin-Hanjani S Tong X Wang J. Extracranial-intracranial bypass and risk of stroke and death in patients with symptomatic artery occlusion: the CMOSS randomized clinical trial. JAMA. (2023) 330:704–14. doi: 10.1001/jama.2023.13390, PMID: 37606672 PMC10445185

[15] Aghaebrahim A Jovin T Jadhav AP Noorian A Gupta R Nogueira RG. Endovascular recanalization of complete subacute to chronic atherosclerotic occlusions of intracranial arteries. J Neurointerv Surg. (2014) 6:645–8. doi: 10.1136/neurintsurg-2013-010842, PMID: 24249733

[16] Chu X Meng Y Zhang J Sun L Yin H Dong K. Safety and efficacy of endovascular recanalization for symptomatic non-acute atherosclerotic intracranial large artery occlusion. Front Neurol. (2023) 14:1144622. doi: 10.3389/fneur.2023.1144622, PMID: 37188310 PMC10176085

[17] Li G Liu P Gong W Zhang X Zhang Y Wang N. Endovascular recanalization for symptomatic intracranial internal carotid and middle cerebral artery occlusion lasting longer than 72 h: experience in a single center. Brain Circ. (2021) 7:259–64. doi: 10.4103/bc.bc_58_21, PMID: 35071842 PMC8757499

[18] Jin M Fu X Wei Y Du B Xu XT Jiang WJ. Higher risk of recurrent ischemic events in patients with intracranial in-stent restenosis. Stroke. (2013) 44:2990–4. doi: 10.1161/STROKEAHA.113.001824, PMID: 23963335

[19] Yin H Zhang J Zhao W Zheng M Song Y Sun L. Drug-coated balloon for the treatment of nonacute symptomatic intracranial carotid artery terminus occlusion: initial experience and follow-up outcome. Front Neurol. (2022) 13:840865. doi: 10.3389/fneur.2022.840865, PMID: 35222260 PMC8879511

[20] Zhang J Zhang X Zhang JP Han J. Endovascular recanalisation with drug coated balloon for chronic symptomatic middle cerebral artery total occlusion. J Neurointerv Surg. (2018) 10:e24. doi: 10.1136/neurintsurg-2017-013693.rep, PMID: 29627791

